# Progress in Medicine

**Published:** 1897-10-02

**Authors:** 


### Progress in Medicine.

### DISEASES OF THE BLOOD.

(*Continued from page 443, Vol. XXII.*)

**Histology.**—The process of making permanent microscopical preparations of the blood involves drying the film of blood and fixing the hæmoglobin. A rapid and easy method is given by C. L. Gulland.[8] He takes a drop and spreads it evenly between two cover-slips, avoiding all pressure with the greatest care. The slips are gently slided off each other and dropped on to the fixing solution, which is made up of 25c.c. of an absolute alcohol solution of eosin (saturated), an equal quantity of pure ether, and five drops of a one in ten solution of corrosive sublimate in absolute alcohol. This is placed in a flat dish, and the cover-slips left in it for three minutes; they are then washed in water and stained, for not more than one minute, in a saturated watery solution of methylene blue, again washed, dipped into absolute alcohol to remove the water and cleared in xylol, and mounted in xylol balsam on a slide. The corpuscles are stained pink, the nuclei blue, and the granules are well brought out, but to obtain success the glasses must be perfectly clean. Giffard,[9] indeed, prefers dry mounting to balsam; but, if anything must be used, oil of cinnamon or naphthalinambar are best. He advises that the slips should be fixed by placing them for two hours in absolute alcohol and ether. Since the importance and increasing value of a study of the blood as a means of diagnosis have been only recently recognised, the writer does well to refer to the mass of practical information given in Cabot's work on "The Clinical Examination of the Blood."

Prout[10] mentions that when leucocytosis is absent in an acute disease we can at once affirm that we are dealing with one of four only, for it is to be found in all others. These four are tuberculosis, malaria, measles, and typhoid. This is of great importance, for instance, in diagnosing typhoid from appendicitis and similar affections. In tuberculosis again, besides the absence of leucocytosis, a differential count of the varieties of the leucocytosis shows that the lymphocytes are much diminished and the polynuclear cells are much increased, and besides there are no nucleated red cells and no poikilocytosis. In cancer the lymphocytes are greatly lessened, the polynuclear cells increased, but there is leucocytosis as well as poikilocytosis and nucleated red cells. This will enable us to distinguish a cancer of the œsophagus from an aneurism of the aorta, where the blood will be found to be normal. Pernicious anæmia is distinguished by the absence of leucocytosis, the great reduction of red cells, and the comparative preservation of the hæmoglobin, together with the presence of nucleated red cells and poikilocytosis, with an excess of lymphocytes and a reduction of polynuclear cells. Pneumonia, except the gravest cases, shows a great leucocytosis chiefly in the polynuclear cells, leucocytosis being defined as any increase of white cells above one to five hundred of the red. We may here remark, for the sake of reference, that the varieties of white cells are present in normal blood to within four per cent. of the followings numbers: (1) lymphocytes, 24 per cent.; (2) large mononuclear, 6; (3) transitional, 3; (4) polynuclear, 65; and (5) eosinophiles, 2. Thus, by the examination of films of blood stained by Gulland's method or Ehrlich's triple stain, an entirely new means of diagnosis in various obscure affections is opened up to us.

Another author, Solomon,[11] adds that the absence of leucocytosis is a valuable diagnostic aid in distinguishing tubercular from simple pleurisy or meningitis, while the crisis in pneumonia is marked by the appearance of eosinophile cells. Thus, in the absence of the special forms, a fall of the thermometer will only be temporary. With regard to this disease, H. Kohn[12] says that in the majority of fatal cases the pneumococcus can be also found in the blood. The production of leucocytosis can be seen in the bone-marrow, at least in certain diseases, where after infection all the cells, and especially the eosinophiles, are increased, and many giant cells are present. Together with this proliferation of cells there is, according to Roger and Josué,[13] absorption of the marrow itself. They found that practically the same process took place both in human beings and in rabbits when either were infected with staphylococci. We must not forget, however, that leucocytosis starts also from lymphadenoid tissue in other situations, such as the glands, which become swollen and reddened, and rapidly multiply the white cells under the influence of various poisons and irritants. Spencer[14] notices that while a moderate leucocytosis is the chief safeguard of the organism, an excessive one has its disadvantages, for it promotes an excessive destruction of both red and white cells. The former thus die before they are efficient as oxygen carriers, and the immature white ones have little protective power.

**Splenic Anæmia in Children** has been ascribed by some observers to rickets, and by others to congenital syphilis. Langford Symes[15] gives as the chief symptoms marked anæmia with puffiness of the face, some œdema of the feet, pyrexia, and diarrhœa, a diminution of red cells and hæmoglobin, a slight increase of white cells, together with an enlarged, painless spleen, hæmorrhages, and the ordinary signs of rickets. It differs then from leucocythemia in the slight increase of white cells it presents, and the greater decrease of red cells. The course of the disease is chronic, and some of the children recover completely. Carr found that 13 out of 30 did so, but Donkin believes the ultimate proquosis is bad. Arsenic, bone marrow, meat juice, and careful diet are recommended as of some use, but the results are slow and unsatisfactory, and the real causation and pathology very obscure. With regard to the hæmorrhages in some of these diseases, it may be useful to remember that Bienwald[16] claims to have stopped the bleeding in a hæmophiliac child by pouring on the wound a little fresh blood derived from a healthy person. He believes that in this way we supply the ferment needed for coagulation, much as A. E. Wright did in similar cases by applying a solution of fibrin ferment from minced glands with calcium chloride. The latter author[17] points out that urticaria, chilblains, and other serous hæmorrhages are often dependent on deficient coagulability, which can be remedied by giving calcium chloride, and avoiding the ingestion of alcohol, sour fruits, and much fluid. Thus, in several cases of chilblains, he found that the time taken by the blood to coagulate was about three times the normal. This slowness of clotting was removed by the careful administration of calcium with the disappearance of the chilblains. It is necessary to test the blood, however, from time to time, lest an overdose should actually increase the slowness of the clot formation. He often gives twenty grains twice or three times daily of the crystallised calcium chloride to commence with, and measures the coagulability by his capillary test tubes.

**Chlorosis.**—In some diseases there is little or no dispute as to the pathology, yet the therapeutic results are nil. Here, on the other hand, the results of treatment are satisfactory in a large percentage of cases, while the pathology is as unsettled as ever. We have a large number of theories of its causation, which are reviewed very fully by C. E. Simon,[18] with the result that most of them are shown to have nothing whatever to do with the question. Thus displacement of the viscera from tight lacing and other causes, as well as gastric atony, are shown by an examination of a long series of cases to be no invariable concomitants. Gastro-intestinal hæmorrhages are proved by chemical tests to be equally unconnected with the disease. Intestinal putrefaction and constipation, so far as their known aromatic products, the sulphates, are concerned, are not found to any marked degree among chlorotics. The author proceeds to summarise the theories of gastric disturbances which have been alleged as causes, and here again exact chemical methods show that none of these derangements is the cause. Thus, if chlorosis be a single disease, none of the theories of it will hold water. He adduces, however, some arguments to show that an insufficient consumption of animal proteids leads to the development of the disease, though the anatomical basis of the disease is undetermined, and urges that the diagnosis should be based on an examination of the blood. If this is done it will be found that the disease is more widespread than is supposed, affecting all ages and both sexes. The treatment by iron does not, he considers, cure a considerable percentage, but better results will attend a diet in which animal proteids, bone-marrow, and dark beer are present. Iron, he thinks, chiefly acts by its stimulating effect on the appetite, for practically the whole amount given can be recovered from the fæces. His argument, that generally there is a subnormal elimination of nitrogen, and therefore a deficient ingestion of nitrogenous food must have occurred, is a doubtful one; and, if so, his positive conclusions are not altogether to be relied on. Bardet[19] remarks that the failure of iron preparations may depend on co-existing peculiarities of digestion. He advocates the hæmoglobin preparations as giving more iron to the organism than others, and as being well borne by the stomach. Robin holds that in some patients oxidation is diminished, and in others it is increased. In the former, iron will remedy the defect; but in the latter arsenic will give better results, as it moderates the nitrogenous changes. Huchard[20] thinks iron harmful in the first stage, which yields to rest and careful diet, as well as in dyspeptics; but this, as Le Gendre remarks, is too dogmatic. The theoretical distinction does not agree with the practical results. Finally, W. H. Porter[21] and others of his school insist that iron is only taken up by the system as a nucleo-albumin from the food, and that the use of iron as a drug is only to shield that contained in these compounds from the sulphur present in the intestines. He finds then three types of anæmia, that from insufficient food, that from the excess of sulphur in the intestines, and that where toxins and other disease products prevent the utilisation of the nucleo-albumin after it has been absorbed. But again we may point to various experiments showing the actual absorption of administered iron. Galley Blackley,[22] in a valuable lecture on the symptoms of chlorosis, advocates the protoxalate of iron, and, where this fails, manganese. Patrin[23] agrees with Robin that the indication for giving iron is to be found when the elimination of nitrogen in the urine has fallen from 80 to 75. Perhaps we may express this by saying that, when the urea becomes scanty, we may give iron with the expectation of more benefit than can be derived from rest and diet alone.

[8] B. Med. Jour., March 13. [9] Med. Record, Feb. 13. [10] Amer. Med. Sur. Bulletin, Feb. 25. [11] Charlotte Med. Rev., Feb. [12] B. Med. Jour., May 15. [13] Med. Week, Dec. and April 2. [14] Lancet, Mar. 6. [15] Dublin Med. Rev. [16] Lancet, July 10. [17] Lancet, July 30. [18] Amer. Jour. Med. Sc., April. [19] Med. Week, April 2. [20] Med. Week, March 3. [21] N. York Med. Jour., May 15. [22] Monthly Hom. Rev., July 1. [23] Les Nouv. Remèdes, p. 423, 1897.

